# How a GNSS Receiver Is Held May Affect Static Horizontal Position Accuracy

**DOI:** 10.1371/journal.pone.0124696

**Published:** 2015-04-29

**Authors:** Steven A. Weaver, Zennure Ucar, Pete Bettinger, Krista Merry

**Affiliations:** Warnell School of Forestry and Natural Resources, 180 E. Green Street, University of Georgia, Athens, Georgia, United States of America, 30602; University of California Los Angeles, UNITED STATES

## Abstract

The static horizontal position accuracy of a mapping-grade GNSS receiver was tested in two forest types over two seasons, and subsequently was tested in one forest type against open sky conditions in the winter season. The main objective was to determine whether the holding position during data collection would result in significantly different static horizontal position accuracy. Additionally, we wanted to determine whether the time of year (season), forest type, or environmental variables had an influence on accuracy. In general, the F4Devices Flint GNSS receiver was found to have mean static horizontal position accuracy levels within the ranges typically expected for this general type of receiver (3 to 5 m) when differential correction was not employed. When used under forest cover, in some cases the GNSS receiver provided a higher level of static horizontal position accuracy when held vertically, as opposed to held at an angle or horizontally (the more natural positions), perhaps due to the orientation of the antenna within the receiver, or in part due to multipath or the inability to use certain satellite signals. Therefore, due to the fact that numerous variables may affect static horizontal position accuracy, we only conclude that there is weak to moderate evidence that the results of holding position are significant. Statistical test results also suggest that the season of data collection had no significant effect on static horizontal position accuracy, and results suggest that atmospheric variables had weak correlation with horizontal position accuracy. Forest type was found to have a significant effect on static horizontal position accuracy in one aspect of one test, yet otherwise there was little evidence that forest type affected horizontal position accuracy. Since the holding position was found in some cases to be significant with regard to the static horizontal position accuracy of positions collected in forests, it may be beneficial to have an understanding of antenna positioning within the receiver to achieve the greatest accuracy during data collection.

## Introduction

Since the introduction of global navigation satellite systems 30 years ago, global navigation satellite system (GNSS) receivers have become a popular tool in natural resource management. Their integration has been somewhat slower in forestry because of difficulties in acquiring quality satellite signals under forest canopies [1], but in general, this technology is steadily replacing traditional navigation and mapping techniques [2]. GNSS receivers can be used for a variety of field work tasks. For example, they can be used for navigation, to locate permanent field plots, to map ownerships or management unit boundaries for use in geographical information systems (GIS), or to map points of interest for management or research. They are also frequently used in wildlife management research to track and locate GNSS-tagged wildlife. A number of recent studies have been conducted to evaluate the static horizontal position accuracy of GNSS receivers in forestry applications [3,4,5,6,7]. While GNSS receivers have been shown to provide fairly accurate location information, several studies have found that vegetation type and canopy cover can have a significant effect on location accuracy [4,8,9,10]. Other factors that may affect location accuracy have been tested including collecting data points during different seasons (i.e., summer or leaf-on vs. winter or leaf-off), and under different environmental conditions such as varying air temperature and humidity [2,5,6]. Additional research has been conducted to identify any variation in accuracy that may result from post-process differential correction [4,8,9]. As the desire for highly accurate location data increases and GNSS technology changes, these receivers need to be continually reassessed to provide natural resource managers with a better understanding of the accuracy of this technology and the factors that influence positional accuracy [2].

GNSS data accuracy can be assessed in two general ways: through horizontal accuracy and through vertical accuracy. Vertical accuracy involves comparing GNSS position fixes collected over a known control point, at a specific height above ground. Horizontal accuracy is generally assessed by static position and dynamic (kinematic) position analyses. Dynamic analysis is so named because the user is typically moving while collecting position fixes, such as when a user walks a boundary line to map an area feature [11]. Static horizontal position accuracy assessments are the most common accuracy analyses, and this type of analysis will be the focus of this study. A static horizontal position accuracy assessment is generally performed by the user holding the GNSS receiver over a known control point and collecting position fixes. These are then compared to the known control point coordinates to estimate accuracy.

GNSS receivers can generally be divided into three categories: recreational-grade, mapping-grade, and survey-grade. Recreational-grade receivers typically range in price from $100 to $700 USD. Wing [12] reported a static horizontal position accuracy range for such receivers of 5–10 m, depending on environmental conditions, with the best performing receivers capable of accuracies within 2 m under open-canopy conditions. In a long-term study [2], static horizontal position accuracy of one type of recreation-grade receiver was found to be 7–10 m, depending on the forest type. Mapping-grade receivers generally cost in the range of $1,000 to $9,000 USD and are typically provide higher static horizontal position accuracy than recreational-grade receivers, in the range of 2–5 m. Most mapping-grade receivers are small enough to be hand-held but are not quite as compact as recreational-grade receivers. Survey-grade receivers are the most expensive type, typically cost $10,000 USD or more, but provide the highest level of positional accuracy. Generally, they are able to provide sub-meter to centimeter levels of static horizontal position accuracy. They are typically only used for property surveys since these receivers are not as portable as mapping-grade or recreational-grade receivers, and they are usually positioned over sample points from several minutes to several hours at a time to attain these levels of accuracy [13]. Mapping-grade and recreational-grade receivers have become most common in forestry applications due to cost, desired accuracy, and mobility. Recommendations on the type of receiver to utilize should be based on the desired level of accuracy and cost [2,3].

As noted previously, as GNSS technology improves and new receivers are placed on the market, a continued assessment of their accuracy seems necessary. The goal of this work was to examine the static horizontal position accuracy of a relatively new GNSS receiver, a F4Devices Flint. This receiver was chosen for this study because of its relatively new design. We evaluated variation in static horizontal position accuracy based on a variety of environmental factors, including season and forest type. From personal communication with a Flint distributor, the orientation of the GNSS antenna within the receiver may affect static horizontal position accuracy. Of all the studies reported in the literature thus far, receiver orientation or holding position during data collection has not been mentioned or studied. The following hypotheses were tested via two field studies:

1. Horizontal position accuracy is not affected by receiver orientation during data collection.
2. Horizontal position accuracy is not affected by season of data collection.
3. Horizontal position accuracy is not affected by forest type.
4. Horizontal position accuracy is not affected by local environmental variables.
5. Horizontal position accuracy is not affected by forest structure obstructions.
6. Horizontal position accuracy does not improve with greater numbers of position fixes used to determine a horizontal position on the ground.

Therefore the overall aim of the work was to determine whether the holding position during data collection would result in significantly different static horizontal position accuracy, and whether the time of year (season), forest type, or environmental variables had any influence on accuracy. Our two studies effectively offer insight into these issues with respect to a currently-available mapping-grade GNSS receiver.

## Materials and Methods

**Fig 1:**
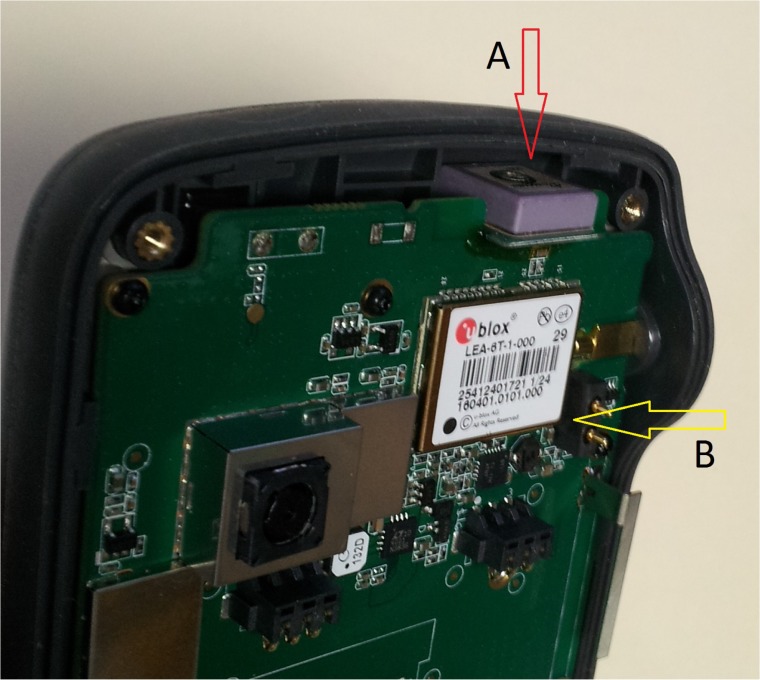
Cirocomm antenna (A) and u-blox receiver (B) within the Flint GNSS receiver.

For this project, we evaluated the static horizontal position accuracy of one type of GNSS receiver, a mapping-grade F4Devices Flint S-series (S812) receiver. The receiver utilizes touch-screen technology through Windows 6.5 Classic, is relatively light-weight (307 grams), can be used when temperatures are between -20°C and 60°, and is considered a rugged device [14]. The receiver has navigation, waypoint and track mapping functions, and advanced data collection capabilities through a variety of software add-ons. The GNSS receiver has 50 channels, and can acquire signals using the GNSS L1 frequency, available through the Coarse / Acquisition (C/A) code of the NAVSTAR (United States) global navigation service, along with the comparable L1 code from the GLONASS (Russian) global navigation service. The time required to acquire the first position fix, from a cold or warm starting condition, is reported to be 29 seconds [14]. The receiver employs a Cirocomm active antenna [15] and a u-blox LEA-6 series receiver module [16] to determine positions. The Cirocomm active antenna lies perpendicular to the normal orientation of the device (Fig 1), while the u-blox receiver lies parallel to the normal orientation of the device. While impedance may vary due to items surrounding the antenna and the position of the antenna board or ground plane, this type of Cirocomm device seems to have been manufactured with the appropriate impedance values to match the design of the overall GNSS receiver [17]. A characteristic diagram of the GNSS antenna impedance can be found in [18]. Information available from the United States Federal Communications Commission [19] regarding the ZK7SSERIES equipment (the Flint GNSS receiver), submitted by BAP Precision Ltd. (the manufacturer) is limited to the radiation pattern and tests of the GSM (cellular phone), Wi-Fi, and Bluetooth antenna components of the Flint GNSS receiver (all optional) that relate to radio frequency (RF) exposure to humans. Unfortunately, an explicit description of the directional horizontal-plane pattern (the gain in reception versus the azimuth angle in the horizontal plane, or gain pattern [20]) of the GNSS antenna, the efficiency of reception with regard to receiver position, and the pattern shape of the peak response are all elusive pieces of information for the receiver tested. While this information would be of value to this study, some indication of the response of carrier-to-noise density (C/N_o_) with respect to the elevation angle of the antenna is observable in the results of this study.

### Study 1: Season and Forest Type Comparison

When collecting GNSS data, field personnel may encounter a variety of field conditions. Since seasonal variations in accuracy were of interest, the GNSS receiver was tested in leaf-off (January 19—February 2, 2013) and leaf-on (May 21–24, 2013) vegetation conditions, and within two different stand types (older deciduous and older coniferous) in order to examine potential differences. Six control points were chosen from the Whitehall Forest GNSS Test Site in Athens, GA, U.S.A. The test site was established in 2004 and is based on a set of survey monuments established using an Ashtech Locus survey-grade GNSS receiver with the appropriate protocols (static data, 4 hours of data collection, etc.) in order to be accepted as National Spatial Reference System (NSRS) positions. The NSRS monuments were processed using the U.S. Department of Commerce, National Oceanic and Atmospheric Administration's Online Positioning User Service (OPUS) (www.ngs.noaa.gov/OPUS), and the positional precision was stated to be less than 2 cm. The closed traverse network that represents the control points within the Whitehall Forest GPS Test Site was subsequently established by registered surveyors using a Topcon GTS-211D instrument, using the NSRS monuments as a base. The closure of the closed traverse network was estimated to be 1/92,137. The control points within the traverse therefore have surveyed horizontal positions that are known to within about 2 cm making the control points within the Test Site a highly accurate model around which GNSS equipment could be tested.

**Fig 2:**
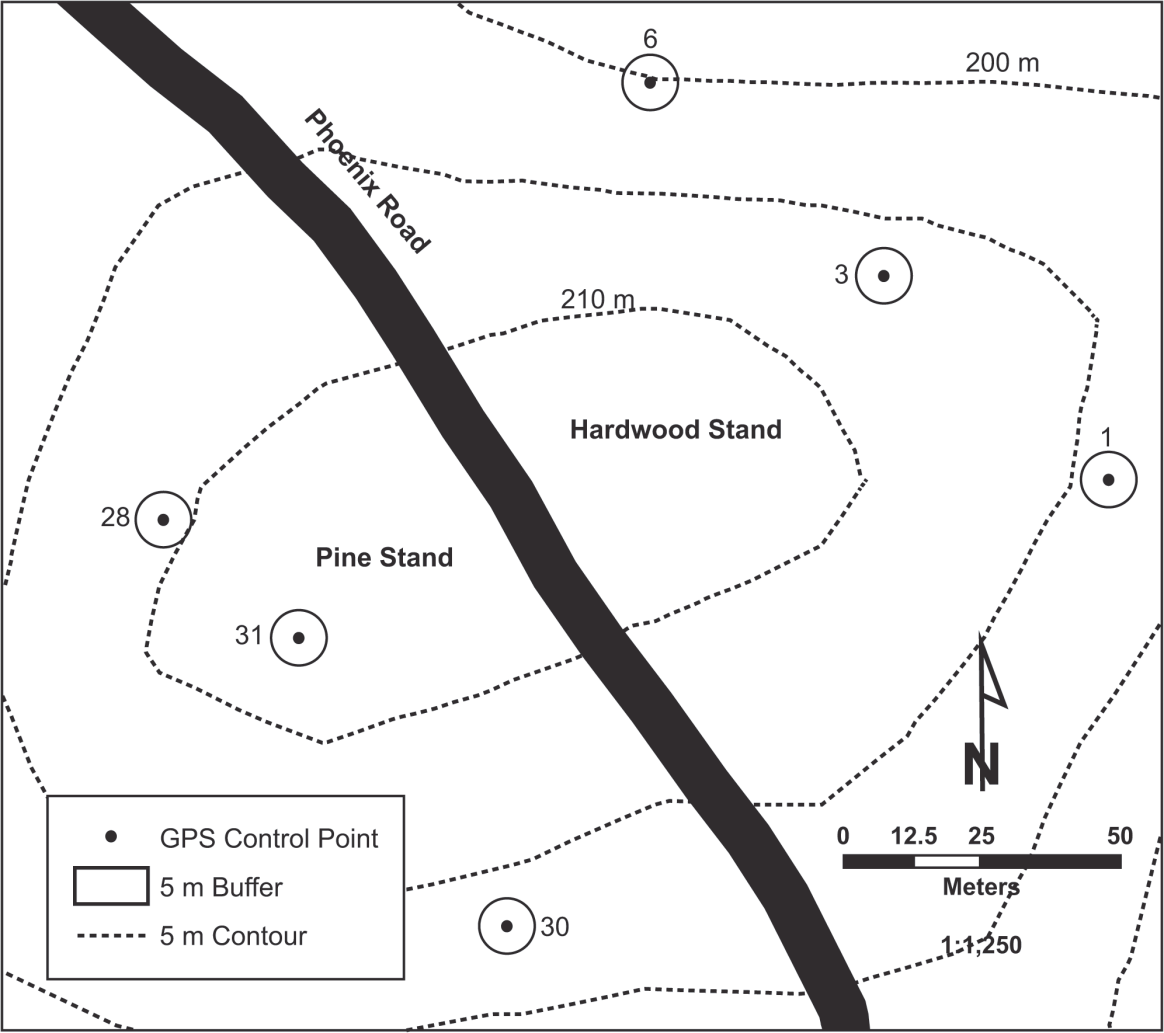
The surveyed control points, with a 5 m buffer shown, located at the Whitehall Forest GPS test site in Athens, Georgia, U.S.A.

Three control points were located within an older coniferous (*Pinus echinata*, *Pinus taeda*) stand of trees (60 to 70 years old, 22.9 m^2^ ha^-1^ basal area, 303.4 trees ha^-1^), and three control points with a similar topographical position were located within an older deciduous (*Quercus* spp., *Carya* spp., *Ostrya virginiana*, and others) stand of trees (60 to 70 years old, 26.2 m^2^ ha^-1^ basal area, 421.7 trees ha^-1^) (Fig 2). All six control points were visited 10 times, resulting in 30 visits to the coniferous stand and 30 visits to the deciduous stand for each season.

Within each season, visits between forest types and points within each forest type were randomized to avoid bias. For each visit, the GNSS receiver was positioned atop a 1.2 m wooden staff directly above the control point using a plumb bob, while the researcher stood on the North side of the point during data collection. Effort was made to ensure the internal antenna was positioned directly above the control points during data collection. Each day of data collection the GNSS receiver was allowed to warm-up (approximately 5 min) to ensure enough satellites were available for use. The receiver was set to receive the wide area augmentation system (WAAS) satellite signal. At each visit, 50 position fixes per point were collected at 2-second intervals. This process was completed with an automatic function on the Flint GNSS receiver. A range of position fixes have been suggested for estimating GNSS accuracy in previous literature. Over a decade ago it was suggested that 300 position fixes should be used for tests under forest canopy [21]. A later study [22] found that one position fix may not be significantly different than an average of 300. Yet one study [4] found static horizontal position accuracy increased as the number of position fixes increased from 1 to 30 when testing a mapping-grade receiver, and another [5] suggested that a minimum of 50 position fixes were necessary to provide an accurate position in forested conditions. Static horizontal position accuracy has also been found to be about the same when one position fix is used for a point as it is when an average of 60 position fixes are used [9]. Finally, in a study utilizing a recreational-grade receiver [13], it was suggested that the first position fix may provide a position that is not significantly different than an average of the first 50. In considering these findings, we chose to collect 50 position fixes per point visit.

**Fig 3:**
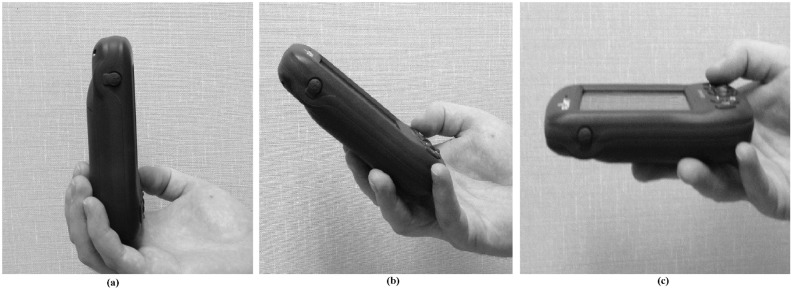
Examples of the three Flint GNSS receiver holding positions used in this study; (a) vertical, (b) angled, and (c) horizontal.

The holding position of the Flint GNSS receiver during data collection was also an area of interest during this study. Because of the design of the Flint unit and the orientation of the antenna within it (Fig 1), the orientation during data collection may result in more accurate data. To test this hypothesis, three holding positions were used: vertical, angled (approximately 45°), and horizontal (Fig 3). The holding position tested was randomized to avoid bias. The sampling order (stand type and control points) was also randomized. Data was collected for 30 visits per season for the Flint GNSS receiver (10 visits to each forest type for each holding position).

### Study 2: Forested and Open Condition Comparison

In the second study, it was of interest to compare results from data collected in forested conditions to results from data collected in an open, unobstructed view condition. The GNSS receiver was only tested in leaf-off (December 7–13, 2014) vegetation conditions, and only one stand type (older coniferous) was considered in order to examine potential differences. The same three older pine control points were selected from the Whitehall Forest GPS Test Site for this test, along with one of the NSRS survey monuments (open sky, unobstructed by trees down to about 10° elevation) that was established in 2004 using the Ashtech Locus survey-grade GNSS receiver. All three control points in the coniferous stand were visited 10 times, resulting in 30 total visits. Positional data was simultaneously collected with a second identical Flint GNSS receiver situated over the NSRS survey monument, in order to facilitate a direct comparison of data collected in forested and open conditions using the same satellite availability.

Visits within the control points in the coniferous stand were randomized to avoid bias. For each visit, the GNSS receiver was positioned atop a 1.2 m wooden staff directly above the control point using a plumb bob, while the researcher stood on the North side of the point during data collection. A similar arrangement was used for data collected over the NSRS survey monument. Effort was made to ensure the internal antenna was positioned directly above the control points during data collection. The GNSS receivers were allowed to warm-up (approximately 5 min) to ensure enough satellites were available for use. The receivers were set to receive the wide area augmentation system (WAAS) satellite signal. At each visit, 50 position fixes per point were collected at 2-second intervals. This process was completed with an automatic function within the Flint GNSS receivers. As in the first study, three holding positions were used: vertical, angled (approximately 45°), and horizontal (Fig 3). The holding position tested was randomized to avoid bias, but was simultaneously employed in the forested and open conditions.

### Statistical Tests

Root mean square error (RMSE) was chosen to evaluate the static horizontal position accuracy as it has been a useful measure to assess the accuracy of GNSS receivers in previous studies. There are two possible ways of calculating RMSE: (1) calculate the squared error for the set of position fixes at each control point for each visit and determine the square root of the mean squared error, or (2) average the set of position fixes and calculate the square root of the squared error of this value. For the two studies we chose the first method to report the RMSE. The RMSE values for each visit to each control point were used to evaluate our hypotheses. We also report circular error probable 50 (CEP50) which represents the radius of a circle around the known control point in which 50% of the position fixes occur, or in other words, the median error within each set of position fixes.

In addition to position coordinates, the Flint GNSS receivers also recorded horizontal dilution of precision (HDOP), positional dilution of precision (PDOP), satellites used, and C/N_o_ values for each position fix. C/N_o_ represents the ratio of carrier power to noise power in a 1-Hz bandwidth [23] and provides an indication of the signal power of a tracked satellite and the noise density viewed by the receiver. It is a term often used interchangeably with signal-to-noise ratio, although technically they are different. These values are of interest because they relate to satellite availability, signal quality, and satellite geometry quality. The values for each fix were then used to calculate averages for each point visit. Air temperature, relative humidity, and barometric atmospheric pressure data were also obtained for each point visit from the local weather station (Athens, Georgia airport). These variables were chosen because of their potential influence on the GNSS signal as it passes through the atmosphere. The weather station only reported these values in one hour intervals. Therefore, a linear change was assumed between hourly observations from the weather station so values might be obtained for each point visit time.

Hypotheses 1–4 relate to the first study, hypotheses 1 and 5 relate to the second study, and hypothesis 6 relates to both studies. To address hypothesis 1, holding positions of the Flint GNSS receiver were compared within each stand and within each season. To address hypothesis 2, no significant difference in accuracy between seasons, coniferous winter values vs. coniferous summer values and deciduous winter values vs. deciduous summer values were compared. Coniferous winter values vs. deciduous winter values and coniferous summer values vs. deciduous summer values were compared to test for significant differences in accuracy between forest types or hypothesis 3. To address hypothesis 4, a Pearson correlation analysis was performed between the RMSE values and mean HDOP, PDOP, vertical dilution of precision (VDOP), satellites used, C/N_o_, air temperature, relative humidity, and atmospheric pressure values. VDOP was derived from HDOP and PDOP values reported by the GNSS receiver [24]. To address hypothesis 5, coniferous winter RMSE values vs. unobstructed open site RMSE values were compared to test for significant differences. Finally, to address hypothesis 6, the first position fix collected from each visit to each control point was compared against the average of the first 10, 20, 30, 40, and 50 position fixes collected to test for significant differences. The normality of the sets of RMSE values used in these tests was evaluated using BestFit software [25]. The majority of the data sets were normally distributed; therefore, a Student’s *t*-test was used to compare the data sets related to hypotheses 1, 2, 3, 5 and 6.

## Results

### Study 1: Season and Forest Type Comparison

With regard to the first study, the mean RMSE values observed for the Flint GNSS receiver in the deciduous stand were in the 3 to 5 m range (Table 1). However, the vertical receiver position had a noticeably lower mean RMSE value in both seasons, as much as 1.5 m lower on average. Mean CEP50 possessed a similar pattern. Both PDOP and C/N_o_ showed very little variation throughout the sampling period. PDOP only ranged from 1.8 to 2.0, and C/N_o_ remained in the range of 30.9 to 32.6 in the deciduous stand. PDOP and C/N_o_ were better during the leaf-off period, perhaps due to signal attenuation problems related to a lower amount of vegetation (leaves) during the winter season. Mean air temperature, relative humidity, and barometric atmospheric pressure values recorded for the sampling period are shown in Table 2. The leaf-on season had temperatures in the 26–27°C range with 55–57% humidity on average. Leaf-off temperatures were around 11°C with 35–36% humidity. Atmospheric pressure for both seasons ranged between 1,016 and 1,026 mb.

**Table 1:** Mean static horizontal position accuracy, PDOP, and signal-to-noise values for the Flint GNSS receiver in the deciduous stand (*n* = 30).

Status	Mean	Mean	Mean	Mean
	RMSE ^a^	CEP50 ^b^	PDOP ^c^	C/N_o_ ^d^
	(m)	(m)		(dB-Hz)
*Leaf-on conditions*				
Vertical holding position	3.8	3.7	2.0	32.6
Horizontal holding position	4.6	4.7	1.8	30.9
Angled holding position	4.6	4.6	1.8	31.5
*Leaf-off conditions*				
Vertical holding position	3.4	3.4	1.8	33.4
Horizontal holding position	4.5	4.6	1.9	31.0
Angled holding position	5.0	5.0	1.9	32.0

^a^ Root mean squared error

^b^ Circular error probable 50

^c^ Positional dilution of precision

^d^ Carrier-to-noise density

**Table 2:** Mean environmental conditions during data collection with the Flint GNSS receiver in the deciduous stand.

Status	Mean airtemperature (°C)	Mean relative humidity (%)	Mean atmospheric pressure (mb)
*Leaf-on conditions*			
Vertical holding position	26.9	55	1,015
Horizontal holding position	26.6	57	1,016
Angled holding position	26.6	56	1,016
*Leaf-off conditions*			
Vertical holding position	11.2	36	1,026
Horizontal holding position	11.3	35	1,026
Angled holding position	11.2	36	1,026

A similar pattern in static horizontal position accuracy was observed for the Flint GNSS receiver when used in the coniferous stand (Table 3). Mean RMSE was in the range of 3 to 4 m. The vertical receiver position also had a lower mean RMSE in the coniferous stand, as much as 1 m more accurate. PDOP values were in a similar range (1.7 to 2.1) as the deciduous stand, but the C/N_o_ values were a little higher (32.6 to 34.4). Again, these results may perhaps be due to signal attenuation problems related to a lower amount of vegetation (needles) during the winter season. The environmental variables for the coniferous stand are shown in Table 4. Since data were collected for both stands on the same days, the environmental conditions are very similar to those for the deciduous stand.

**Table 3:** Mean static horizontal position accuracy, PDOP, and signal-to-noise values for the Flint GNSS receiver in the coniferous stand (*n* = 30).

Status	Mean RMSE ^a^ (m)	Mean CEP50 ^b^ (m)	Mean PDOP ^c^	Mean C/N_o_ ^d^ (dB-Hz)
*Leaf-on conditions*				
Vertical holding position	3.1	3.2	1.8	34.4
Horizontal holding position	4.1	4.2	1.9	33.1
Angled holding position	3.9	4.1	2.0	34.2
*Leaf-off conditions*				
Vertical holding position	3.1	3.1	1.7	33.5
Horizontal holding position	3.8	3.8	1.9	32.6
Angled holding position	3.8	3.8	2.1	33.6

^a^ Root mean squared error

^b^ Circular error probable 50

^c^ Positional dilution of precision

^d^ Carrier-to-noise density

**Table 4:** Mean environmental conditions during data collection with the Flint GNSS receiver in the coniferous stand.

Status	Mean air temperature (°C)	Mean relative humidity (%)	Mean atmospheric pressure (mb)
*Leaf-on conditions*			
Vertical holding position	26.8	56	1,015
Horizontal holding position	26.5	58	1,016
Angled holding position	26.5	57	1,016
*Leaf-off conditions*			
Vertical holding position	11.1	36	1,026
Horizontal holding position	11.3	35	1,026
Angled holding position	11.1	36	1,026

The vast majority of the static horizontal position accuracy data for Study 1 were normally distributed, thus no transformations were applied and a Student’s *t*-test was used to determine any significant differences. Using an alpha (*α*) value of 0.05, only one test for the Flint GNSS receiver was found to be significant. The static horizontal position accuracy of data collected during the leaf-off period in the deciduous stand, using a vertical holding position, was found to be significantly different (*p* = 0.012), with greater positional accuracy, than data collected using an angled holding position. Although no other tests were found to be significant when *α* = 0.05, several other tests were significant when *α* = 0.10. One test found significantly better static horizontal position accuracy in forest type (coniferous stand compared to deciduous stand using an angled holding position) during the leaf-off season (*p* = 0.081). Three tests found the vertical holding position static horizontal position accuracy to be significantly different, with greater horizontal position accuracy, than horizontal or angled holding positions, regardless of season or forest type (*p* = 0.058 to 0.092).

With respect to hypothesis 1, during the leaf-off season only one of six potential tests (deciduous forest, vertical versus horizontal holding position) suggested that the hypothesis could be rejected. During the leaf-on season, only two of six potential tests (coniferous forest, vertical versus horizontal holding position and vertical versus angled holding position) suggested that the hypothesis could be rejected. With regard to the vertical holding position and its comparison with the other two holding positions, only 3 of 12 tests suggested the hypothesis could be rejected. Therefore there is weak to moderate evidence that holding position of the GNSS receiver does affect static horizontal position accuracy.

With respect to hypothesis 2, none of the tests performed suggested that the hypothesis could be rejected. Therefore there is no evidence that time of year (season) affects static horizontal position accuracy. With respect to hypothesis 3, only one of the statistical tests (coniferous versus deciduous when using an angled holding position) suggested that the hypothesis related to forest type could be rejected, therefore there is little evidence that forest type (when time of year is constant) affects static horizontal position accuracy.

The *r* values associated with the correlation results were typically in the range of -0.4 to 0.4 (Tables 5 and 6), suggesting weak correlation or none at all. For most of the comparisons there does not seems to be very consistent results. Some relationships have a significant positive correlation values (e.g., relative humidity with RMSE values from holding the unit in an angled position in the coniferous stand during the leaf-on season) which were significant (*p* < 0.05), while other very similar pairs of data had relationships that reflected a negative correlation (holding the unit vertically) or an insignificant correlation (holding the unit horizontally). The inconsistency of the results suggests that the significant relationships may have been observed purely through chance. As a result, we feel unable to reject hypothesis 4, where we stated that static horizontal position accuracy is not affected by local environmental variables.

**Table 5:** Correlation between static horizontal position accuracy (as expressed by the root mean squared error of 30 samples) and other metrics of interest when using the Flint GNSS receiver in the coniferous stand.

Status / Metric	Holding position					
	Vertical		Angled		Horizontal	
	r	*p*-value	r	*p*-value	r	*p*-value
*Leaf-on conditions*						
HDOP ^a^	-0.247	0.188	-0.145	0.446	0.018	0.924
VDOP ^b^	-0.101	0.597	-0.020	0.916	0.172	0.364
PDOP ^c^	-0.142	0.454	-0.048	0.801	0.138	0.467
Satellites used	-0.109	0.565	0.301	0.106	-0.110	0.563
C/N_o_ ^d^	-0.266	0.156	-0.233	0.216	0.053	0.782
Air temperature	0.009	0.946	0.177	0.350	-0.202	0.284
Relative humidity	-0.106	0.576	0.508 ^**^	0.004	0.162	0.393
Atmospheric pressure	-0.038	0.843	-0.012	0.950	0.011	0.955
*Leaf-off conditions*						
HDOP ^a^	0.168	0.376	0.238	0.205	0.347 ^*^	0.060
VDOP ^b^	0.298	0.109	0.259	0.166	-0.099	0.603
PDOP ^c^	0.258	0.169	0.282	0.131	0.016	0.933
Satellites used	-0.187	0.323	-0.206	0.275	-0.106	0.578
C/N_o_ ^d^	0.324 ^*^	0.080	-0.092	0.629	-0.245	0.193
Air temperature	-0.197	0.296	0.018	0.925	0.123	0.519
Relative humidity	0.218	0.247	-0.003	0.989	-0.217	0.250
Atmospheric pressure	0.124	0.515	0.091	0.632	-0.100	0.598

^**^ Significant at *p* < 0.05 level

^*^ Significant at *p* < 0.10 level

^a^ Horizontal dilution of precision

^b^ Vertical dilution of precision

^c^ Positional dilution of precision

^d^ Carrier-to-noise density

**Table 6:** Correlation between static horizontal position accuracy (as expressed by the root mean squared error of 30 samples) and other metrics of interest when using the Flint GNSS receiver in the deciduous stand.

Status / Metric	Holding position					
	Vertical		Angled		Horizontal	
	r	*p*-value	r	*p*-value	r	*p*-value
*Leaf-on conditions*						
HDOP ^a^	0.019	0.919	-0.127	0.503	-0.206	0.275
VDOP ^b^	0.025	0.895	0.069	0.718	0.114	0.548
PDOP ^c^	0.024	0.900	0.007	0.971	0.061	0.749
Satellites used	-0.234	0.213	-0.156	0.410	-0.087	0.649
C/N_o_ ^d^	-0.010	0.959	-0.237	0.207	0.038	0.843
Air temperature	-0.121	0.524	-0.328 ^*^	0.077	-0.143	0.450
Relative humidity	0.157	0.408	-0.213	0.257	0.401^**^	0.028
Atmospheric pressure	0.143	0.452	0.154	0.417	0.074	0.696
*Leaf-off conditions*						
HDOP ^a^	0.025	0.895	0.060	0.752	0.143	0.449
VDOP ^b^	-0.333 ^*^	0.072	0.066	0.727	-0.194	0.304
PDOP ^c^	-0.262	0.162	0.063	0.741	-0.085	0.655
Satellites used	-0.007	0.970	-0.042	0.826	0.227	0.229
C/N_o_ ^d^	-0.267	0.154	-0.145	0.444	-0.285	0.127
Air temperature	0.058	0.762	0.137	0.470	0.161	0.395
Relative humidity	0.032	0.866	-0.143	0.452	-0.323^*^	0.082
Atmospheric pressure	-0.359 ^*^	0.051	-0.064	0.736	-0.223	0.236

^**^ Significant at *p* < 0.05 level

^*^ Significant at *p* < 0.10 level

^a^ Horizontal dilution of precision

^b^ Vertical dilution of precision

^c^ Positional dilution of precision

^d^ Carrier-to-noise density

Often the issue of the appropriate number of position fixes to use arises, and in this study we found no difference at all between the use of one position fix and the average of the first 10, 20, 30, 40, or 50 position fixes. In a few cases, the average of 10 position fixes produced a slightly better static horizontal position accuracy than a single position fix, but in most cases the mean RMSE and CEP50 values became slightly worse as more position fixes were collected. The main exception was for the vertical holding position during the leaf-off season, where in both forest types the static horizontal position accuracy improved slightly (up to 0.13 m) as the number of position fixes increased. However, these trends were not statistically significant using an alpha (*α*) value of 0.10, and the changes range from 0.1 to 0.8 m from the RMSE of the first position fix to the average of 50 position fixes. From these results, one might conclude that the additional number of position fixes collected had little significant impact on the quality of the results. As a result, we are unable to reject hypothesis 6, where we suggested that static horizontal position accuracy does not improve with greater numbers of position fixes.

### Study 2: Forested and Open Condition Comparison

With regard to the second study, the results for the Flint GNSS receiver when used in the coniferous stand indicated that the mean RMSE values were in the 3 to 4 m range (Table 7), which was consistent with and very similar to the results from Study 1 (Table 3) conducted nearly a year earlier. As in the previous study, the vertical receiver position produced a noticeably lower mean RMSE value and mean CEP50 value in the coniferous stand. However, the mean RMSE from the vertical holding position was only significantly different than the mean RMSE derived from the horizontal holding position (*p* = 0.048), not the angled holding position (*p* = 0.127). And as in the previous study, both PDOP and C/N_o_ showed very little variation throughout the sampling period. Since the C/N_o_ differences between forested and open conditions are small (around 5 dB), the gain in static horizontal position accuracy evident between the two sites is likely due to other factors.

**Table 7:** Mean static horizontal position accuracy, PDOP, and signal-to-noise values for the Flint GNSS receiver in the second study, under leaf-off conditions (*n* = 30).

Status	Mean RMSE ^a^ (m)	Mean CEP50 ^b^ (m)	Mean PDOP ^c^	Mean C/N_o_ ^d^ (dB-Hz)
*Coniferous forest condition*				
Vertical holding position	3.2	3.2	2.0	35.6
Horizontal holding position	4.2	4.1	2.0	33.6
Angled holding position	3.9	3.9	2.0	35.0
*Open condition*				
Vertical holding position	0.8	0.8	1.9	38.9
Horizontal holding position	1.1	1.1	1.9	37.1
Angled holding position	0.9	0.9	1.9	37.5

^a^ Root mean squared error

^b^ Circular error probable 50

^c^ Positional dilution of precision

^d^ Carrier-to-noise density

With respect to hypothesis 1, and somewhat similar to what we found with Study 1, we feel that there is weak to moderate evidence that holding position of the GNSS receiver does affect static horizontal position accuracy when the GNSS receiver is used in forested conditions. The mean RMSE values for the open condition were around 1 m (Table 7), and the vertical receiver position had a slightly lower mean RMSE value and mean CEP50 than the other two holding positions. However, these results were not significantly different, although the vertical versus horizontal comparison of holding positions (*p* = 0.132) provided the closest reason to suggest potential significant differences. For the open condition, the PDOP was lower and the C/N_o_ was higher than in the data collected in the coniferous stand, results which were to be expected. With respect to hypothesis 1, there is no evidence that holding position of the GNSS receiver does affect static horizontal position accuracy when the GNSS receiver is used in open conditions.

When comparing the performance of the GNSS receiver in open conditions against forested conditions, and with respect to hypothesis 5, we observed a significant difference (*p* < 0.05) in mean RMSE values. As we noted, static horizontal position accuracy is much better when the receiver was used in unobstructed situations than when it was used in the older coniferous forest conditions.

For data collected in the open condition, results suggested that there was no difference at all between the use of one position fix and the average of the first 10, 20, 30, 40, or 50 position fixes. The data collected from the coniferous stand while holding the Flint GNSS receiver in the vertical position indicated that the RMSE and CEP50 improved slightly from using the first position fix through the use of 30 position fixes. After 30 position fixes, there seemed to be no improvement in mean RMSE or CEP50 values. In contrast, while holding the Flint GNSS receiver in the angled or horizontal position, mean RMSE and CEP50 values became slightly worse as more position fixes were collected, with the first position fix (by itself) possessing greater accuracy than the average of a set of position fixes. However, these trends were not statistically significant using an alpha (*α*) value of 0.10, and the changes ranged from 0.1 to 0.3 m from the RMSE of the first position fix to the average of 50 position fixes. Therefore the number of position fixes collected had little significant impact on the quality of the results, and were are unable to reject hypothesis 6, where we suggested that static horizontal position accuracy does not improve with greater numbers of position fixes.

The environmental variables for the second study indicate that the air temperature was 16.1°C on average, the relative humidity was about 25%, and the atmospheric pressure was 1,022 mb on average. Since there seemed to be little to no (or inconsistent) correlation between these values and observed static horizontal position accuracy in Study 1, and since previously published studies [2,7] have suggested little correlation between these and static horizontal position accuracy, the correlation analysis was not performed for the data collected under Study 2.

## Discussion

The static horizontal positions determined by the Flint GNSS receiver in forested conditions had mean RMSE values in the range of 3 to 5 m; the best static horizontal position accuracy observed from a single position fix was 0.04 m, while the worst was 11.6 m. These results were obtained without using post-process differential correction. A study conducted with two other mapping-grade receivers, several years prior to this on the Whitehall Forest GPS Test Site (and using the same control points), found static horizontal position accuracy to be in the range of 1.6 to 2.1 m, again without employing post-process differential correction to the data [7]. For a third type of mapping-grade receiver, another study conducted on the Whitehall Forest GPS Test Site of a mapping-grade receiver [6] found the static horizontal position accuracy to be in the range of 5.6 to 8.9 m, depending on slope position and season. In this case, after post-process differential correction, the static horizontal position accuracy improved to the 2.0 to 3.1 m range. Comparing our results to the results of these previous studies, we suggest that the static horizontal position accuracy achieved using the Flint GNSS receiver seems to be consistent with other assessments, and is therefore within the static horizontal position accuracy range that one might expect of a mapping-grade receiver without employing post-processing differential correction.

The statistical tests employed showed no significant difference in static horizontal position accuracy when using the Flint GNSS receiver during between seasons (leaf-on vs. leaf-off). The different in mean RMSEs between seasons was less than 1 m. This contrasts with other studies that have shown there is a significant difference in static horizontal position accuracy between seasons [5,6]. In one case, forest type did however have a significant effect on horizontal position accuracy. This significance may be attributed to differences in stand structure, such as trees density or canopy cover. However, there was little evidence otherwise that forest type (when time of year is constant) affected static horizontal position accuracy, and interestingly no significant difference in forest type was found in other work [7] conducted on the same site, with similar quality technology. Interestingly, for several aspects of Study 1, across both forest type and season, we found a few significant differences in results obtained using different holding positions. The vertical holding position in a few tests was found to produce significantly higher quality horizontal positions, on the order of 0.7 m to 1.5 m closer to the true positions, than horizontal or angled holding positions.

The second study, which compared data collected within a coniferous forest to data collected in an open area, produced some interesting results. Many GNSS receiver manufacturers suggest that sub-meter static horizontal position accuracy can be obtained through the use of modern equipment and systems. While previous studies of recreation-grade and mapping-grade receivers within forested conditions have generally failed to show this, our study does suggest sub-meter accuracy can be obtained in open areas with the mapping-grade receiver tested, without post-process differential correction. Our study did not confirm with statistical significance that when an observer is in a open area, how a GNSS receiver is held can affect static horizontal position accuracy. However, when used in forested conditions, our study provided weak to moderate evidence that differences in static horizontal position accuracy may be associated with how a receiver is held. This was noted in both studies presented here, and perhaps the effects of multipath and the inability to use certain satellite signals (due to signal blockage) may have contributed to the results. Finally, the number of position fixes to use in determining a horizontal position is an often-debated subject in the literature [4,5], and both studies suggest that the first position fix collected with the Flint GNSS receiver is generally no different than the average of a number of position fixes. This result may only be representative of data collected by the Flint GNSS receiver, as other studies have suggested otherwise (i.e., more position fixes increases position accuracy). One recent study regarding a recreation-grade receiver [13] produced results consistent with our findings. Therefore the notion that the first position fix obtained is of acceptable quality (and no worse than an average of a set of position fixes) might be attributed to advancements in technology of the overall global positioning program (satellites, antennas, receivers, software, etc.).

A number of factors could have affected the results of this study. Although the data was not examined for any influence, the proximity of the researcher to the receiver during the data collection effort, or the receiver to other nearby trees, may have introduced some bias into the results. This is mentioned because one study [26] found nearby trees to have some influence on static horizontal position accuracy, which may also have influenced the data in this study. Control point location may also have been a factor. While the control points were chosen to be as consistent as possible in terms of forest conditions and elevation, there were some differences in aspect. Some points were on more northern and eastern aspects while others were on a more southerly aspect.

There was also no pre-planning or mission planning performed to schedule data collection at times with the best predicted PDOP. We chose not to pre-plan our data collection effort in order to minimize the entire data collection time and to mimic what the average person would do in the field. Collecting data during these times may have provided better static horizontal position accuracy or at the very least, more satellite availability and signal quality, but other responsibilities limited the available times for data collection. WAAS signal availability should also be a consideration. The receiver was programmed to utilize the WAAS signal if it was available, but did not record when the signal was used or what impact it had on these results. One might expect however that static horizontal position accuracy would increase with the use of the WAAS signal. However, in at least one study [4] no statistically significant differences have been noted when WAAS was utilized with mapping-grade receivers. Another concern is the use of only one of each receiver of this type to collect data (Study 1). Further, equal performance among similar receivers is not guaranteed (Study 2) and should not be assumed [27]. Due to time and receiver availability, we were limited in the number of pieces of equipment that could effectively be tested.

## Conclusion

The first study (comparison of seasons and forest types) and its results were consistent in many ways with previous studies that tested mapping-grade receivers. We did not find a significant difference in static horizontal position accuracy due to season with the Flint GNSS receiver. This result may be due to the ability of the Flint GNSS receiver to effectively handle multipath signals that may occur in response to changes in canopy cover. RMSE was found to be significantly different between forest types (greater in the deciduous stand than in the coniferous stand) in only one of the tests conducted. This result could be attributed to differences in forest density and canopy cover. As canopy and stocking conditions change across forest types, changes in static horizontal position accuracy might be expected as well. For example, in forest stands with greater stocking and canopy cover, RMSE might be expected to increase due to greater multipath potential and blocked GNSS signals. However, we found little evidence of this result when using the F4Devices Flint GNSS receiver. With respect to collecting data in forested conditions for typical forestry purposes, the effect of changes in foliage is likely not a major concern for users of technology of this quality. Further, there seemed to be no significant different in the use of one position fix versus the average 50 (or less) position fixes when computing RMSE or CEP50 values, and weak to moderate evidence that the manner in which the GNSS receiver was held may affect static horizontal position accuracy.

The second study (forested versus open conditions) and its results were also consistent in many ways with previous studies that tested mapping-grade and recreation-grade receivers. For example, there seemed to be no significant different in the use of one position fix versus the average 50 (or less) position fixes when computing RMSE or CEP50 values. While we did once again see some significant differences in accuracy arising from the holding position when used in the coniferous stand, there were no significant differences in accuracy arising from the manner in which the GNSS receiver was held when used in the open condition. In sum, for the Flint GNSS receiver using the antenna configuration described earlier, there is only weak to moderate evidence that the effect of holding position on static horizontal position accuracy is significant, and it only seems to be evident when the receiver is used in forested conditions. While from a GNSS perspective, the differences observed when adjusting the holding position may not be significant under other conditions or when using other GNSS receivers, users of GNSS technology should keep this issue in mind and perform tests themselves to ensure data quality.
